# Clinical Efficacy of Stem Cell Therapy for Diabetes Mellitus: A Meta-Analysis

**DOI:** 10.1371/journal.pone.0151938

**Published:** 2016-04-13

**Authors:** Ahmed El-Badawy, Nagwa El-Badri

**Affiliations:** Center of Excellence for Stem Cells and Regenerative Medicine (CESC), Zewail City of Science and Technology, 6^th^ of October City, Egypt; University-Hospital of Parma, ITALY

## Abstract

**Background:**

Stem cell therapy is a promising therapeutic modality for advanced diabetes mellitus (DM). This study presents a meta-analysis of relevant clinical trials to determine the efficacy of stem cell therapy in DM. We aim to critically evaluate and synthesize clinical evidence on the safety and efficiency of different types of stem cell therapy for both T1DM and T2DM.

**Methods and Findings:**

We pooled participant-level data from twenty-two eligible clinical trials that satisfied our inclusion criteria, with a total of 524 patients. There were significant differences in the outcome based on the type and source of the infused cells. Out of all T1DM patients who received CD34^+^ hematopoietic stem cell (HSC) infusion, 58.9% became insulin independent for a mean period of 16 months, whereas the results were uniformly negative in patients who received umbilical cord blood (UCB). Infusion of umbilical cord mesenchymal stem cells (UC-MSCs) provided significantly beneficial outcome in T1DM, when compared to bone-marrow mesenchymal stem cells (BM-MSCs) (P<0.0001 and P = 0.1557). Administration of stem cell therapy early after DM diagnosis was more effective than intervention at later stages (relative risk = 2.0, P = 0.0008). Adverse effects were observed in only 21.72% of both T1DM and T2DM stem cell recipients with no reported mortality. Out of all poor responders, 79.5% were diagnosed with diabetic ketoacidosis.

**Conclusions:**

Stem cell transplantation can represent a safe and effective treatment for selected patients with DM. In this cohort of trials, the best therapeutic outcome was achieved with CD34^+^ HSC therapy for T1DM, while the poorest outcome was observed with HUCB for T1DM. Diabetic ketoacidosis impedes therapeutic efficacy.

## Introduction

According to the International Diabetes Federation, DM affects more than 300 million people worldwide, causing substantial morbidity and mortality [1]. Whole organ or islet transplantation; and especially following the Edmonton protocol, have been few of the most promising therapies for T1DM [2]. However, this procedure suffers many hurdles, including lack of donors and requirement for life-long immune suppression. A single 68 kg (150 lb) patient requires transplantation of roughly 340–750 million islet cells to effectively resolve the disease [3–5]. In clinical practice, this necessitates two or three donors of pancreatic islets for a transplantation procedure into a single patient.

Stem cell therapy represents a highly promising new modality of treatment for advanced diabetes. However, many concerns about the type of stem cells, the transplantation procedure, and long-term recovery remain to be addressed [6]. Numerous *in vivo* animal studies demonstrated the potential advantages of using stem cells to treat DM. However, given the complexity of the treatment and the potential ethical and translational considerations, just a few have moved to the clinic. This systematic review and meta-analysis aims to critically evaluate and synthesize clinical evidence on the safety and efficiency of different types of stem cell therapy for both T1DM and T2DM. We define safety as the absence of adverse events, and efficacy as a significant improvement in pancreatic endocrine function after therapy. This study may help in the design of future clinical trials, and provide guidelines to the concerned community of physicians and patients on the outcome of stem cell therapy in DM.

## Research Design and Methods

### Selection of studies

The screening of eligible publications was carried out independently by the authors; and any discrepancy was resolved by consensus. Eligible studies had to have a minimal follow-up period for at least a 6-months after the initiation of the therapy. Studies in which the subjects had any additional pathologies or altered endocrine status other than DM were excluded.

### Search strategy

An extensive literature review with no language restriction was carried out up to August 2015 across several databases of MEDLINE, EMBASE, Google Scholar, CINHal, Cochrane Central Register of Controlled trials (CENTRAL), Current Controlled Trials (ISRCTN), ClinicalTrials.gov, WHO ICTRP, UMIN-CTR and the Hong Kong Clinical Trials Register. The database was searched using the following key words: (stem cells, progenitor cells, bone marrow) AND (diabetes mellitus, hyperglycemia). We checked the reference lists of all identified eligible papers and relevant narrative reviews.

### Data extraction and assessment of risk of bias

The risk of bias of the extracted data was determined using the inclusion criteria outlined in the *Cochrane Handbook for Systematic Reviews of Interventions (PRISMA)* [7]. Attrition, confounding measurement, intervention, performance, selection and conflict of interest were graded as low risk, high risk and unable to determine (S1 Checklist) [7].

### Statistical analysis

Extracted data were entered into Review Manager Version 5.3 database and GraphPad Prism 6. The statistical reporting was performed according to the previously published guidelines [8] and the guidelines of reporting systematic reviews [9, 10]. The mean values of the C-peptide levels, HbA1C levels, and insulin requirement before and after therapy, or between treated and untreated patients (controls) were compared.

We used weighted mean difference with random effects model to avoid heterogeneity, in line with the previously published guidelines for statistical reporting and the Cochrane Handbook for Systematic Reviews of Interventions [11, 12]. Heterogeneity was considered significant at P<0.10 [13]. Inconsistency was estimated using the I^2^ statistics; values of 25, 50, and 75% were considered low, moderate, and high inconsistencies, respectively [14].

For clinical perspective, stratification analysis was also conducted to examine the impact of several factors such as the cell type [bone marrow hematopoietic stem cells (BM-HSCs), bone marrow mesenchymal stem cells (BM-MSCs), umbilical cord mesenchymal stem cells (UC-MSCs), and adipose stem cells (ASCs)]; number of cells injected (<10^7^ or ≥10^7^); method of cell delivery (intravenous or intrapancreatic administration); and follow up period after stem cell therapy.

## Results

### Search results and description of studies

**Fig 1:**
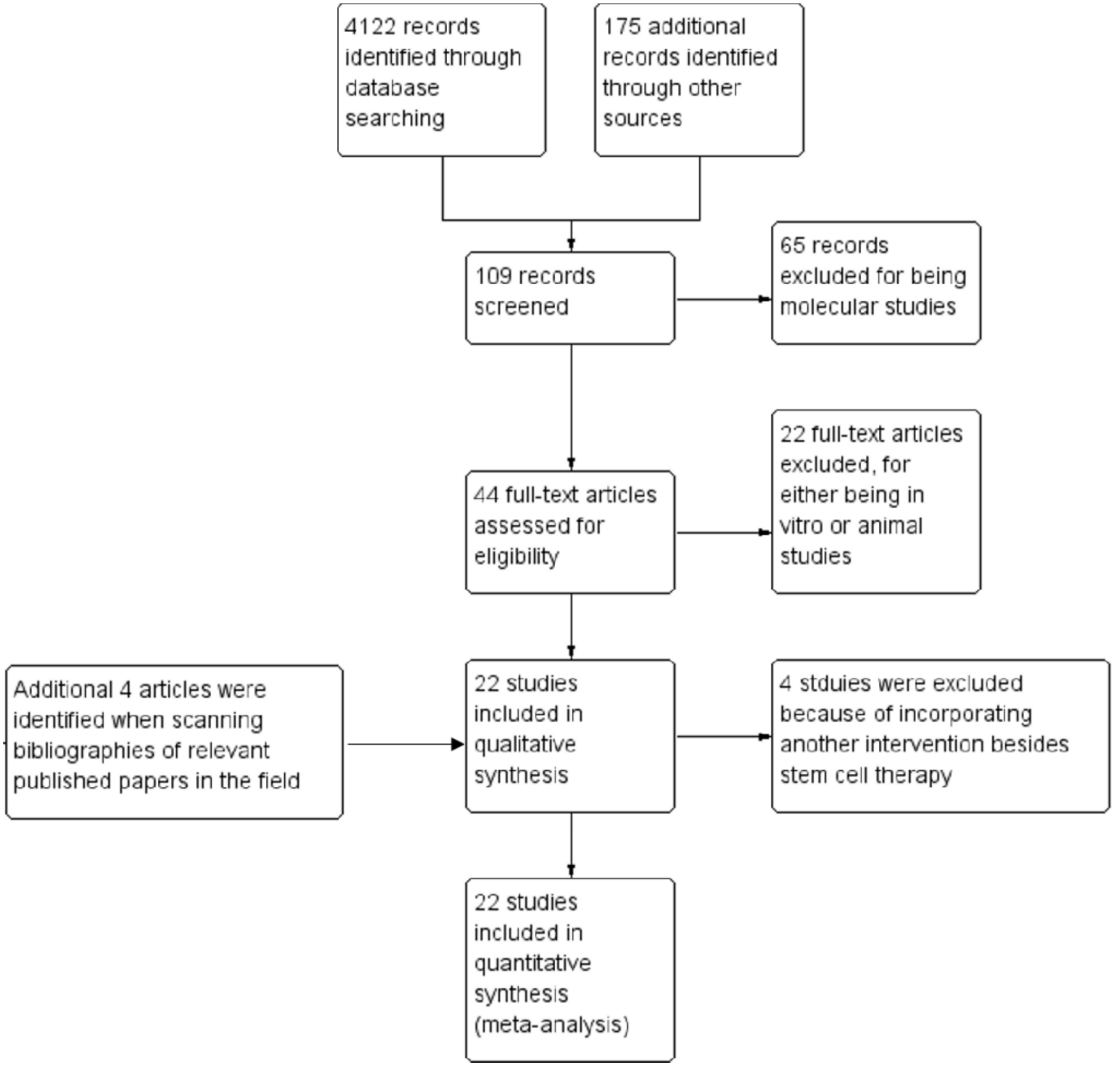
Flow diagram showing record identification, screening and study inclusion process.

After the selection process, 22 eligible clinical trials reporting stem cell-based therapy for DM with a total of 524 patients were included in the present analysis [15–36]. The selection process of the trials is shown in Fig 1. The individual data are listed in Table 1.

**Table 1:** Baseline characteristics of the study populations in the 22 studies included in this analysis.

Author	Number of Patients	Mean Age of Patients (years)	Mean History of Disease	Regimen	Mean Dose of Injected cells/kg	Mode of Injection	Mean Follow Up Period	Reference
Thakkar 2015	20 (T1DM)	19.95	8.1 y	IS-ADMSCs + BM-HSCs	2.07 × 10^4^	Intra-pancreatic	33 m	[32]
Carlsson 2014	20 (T1DM)	24	< 3 weeks	BM-MSCs	2.75 × 10^6^	IV	12 m	[17]
D’Addio 2014	65 (T1DM)	20.4	< 3 m	HSCs	5.8 × 10^6^	IV	48 m	[19]
Haller 2013	15 (T1DM)	6.9	3.9 m	UCB	1.1 × 10^7^	IV	12 m	[23]
Bhansali 2013	21 (T1DM)	51	12 y	BM-MNCs	2.9 × 10^8^	Intra-pancreatic	12 m	[15]
Giannopoulou 2013	17 (T1DM)	4.81	1 y	UCB	1.27 × 10^6^	IV	12 m	[20]
Mesples 2013	3 (T1DM)	7	< 2 m	BM-MNCs	180 × 10^6^	Liver puncture	12 m	[30]
Hu 2013	29 (T1DM)	17.9	New onset	UC-MSCs	2.6 × 10^7^	IV	21 m	[26]
Li 2012	13 (T1DM)	14.10	< 12 m	HSCs	4 × 10^6^	IV	42 m	[28]
Zhang 2012	9 (T1DM)	15.7	2 y	HSCs	10.49 × 10^6^	IV	12 m	[36]
Gu 2012	28 (T1DM)	17.6	3 m	HSCs	-------	IV	19.3	[21]
Haller 2011	24 (T1DM)	5.1	4 m	UCB	1.88 × 10^7^	IV	12 m	[22]
Snarski 2010	8 (T1DM)	25.8	2 y	HSCs	4.14 × 10^6^	IV	6 m	[31]
Vanikar 2010	11 (T1DM)	21.1	8.2 y	IS-ADMSCs + BM-HSCs	3.15 × 10^6^	Intra-pancreatic	23 m	[37]
Couri 2009	23 (T1DM)	18.4	< 3 m	HSCs	10.52 × 10^6^	IV	29.8	[18]
Haller 2009	15 (T1DM)	5.25	4.1 m	UCB	1.5 × 10^7^	IV	12 m	[24]
Liu 2014	22 (T2DM)	52.9	8.7 y	UC-MSCs	1 × 10^6^	IV + intrapancreatic on Day 5	12 m	[29]
Wu 2014	40 (T2DM)	55.9	≥2 to ≤ 15 y	BM-MNCs	382.6 × 10^7^	Pancreatic arterial infusion	12 m	[35]
Tong 2013	3 (T2DM)	41	6.58 y	UCB	2.88 × 10^6^	Intra-pancreatic	6 m	[33]
Hu 2012	118 (T2DM)	50.4	8.6 y	BM-MNCs	2.8 × 10^9^	Intra-pancreatic	33 m	[25]
Jiang 2011	10 (T2DM)	66	11 y	PD-MSCs	1.35 × 10^6^	IV	6 m	[27]
Bhansali 2009	10 (T2DM)	57.5	14.6 y	BM-MNCs	3.1 × 10^6^	Intra-pancreatic	6 m	[16]

**Abbreviations: T1DM**: type 1 diabetes mellitus, **T2DM**: type 1 diabetes mellitus, **BM-HSCs**: bone marrow-hematopoietic stem cells, **BM-MSCs**: bone marrow-mesenchymal stem cells, **BM-MNCs**: bone marrow-mononuclear stem cells, **UCB**: umbilical cord blood, **UC-MSCs**: umbilical cord-mesenchymal stem cells, **PD-MSCs**: placenta derived-mesenchymal stem cells, **IS-ADMSCs**: insulin secreting-adipose derived mesenchymal stem cells, **IV**: Intravenous

The mean patient age was 26.18±19.59 years. Out of the 23 trials, stem cell therapy was evaluated in patients with either T1DM (15 studies, 300 patients) or T2DM (7 studies, 224 patients). Considering the source of cells, 6 studies used HSCs (149 patients) [18, 19, 21, 28, 31, 36], 5 studies used BM-mononuclear cells (BM-MNCs) (189 patients) [15, 16, 25, 30, 35], 5 studies used UCB (74 patients) [20, 22–24, 33], 2 studies used UC-MSCs (51 patients) [26, 29], 2 studies used a combination of different stem cells (31 patients) [32, 37], one study used BM-MSCs (20 patients) [17], and one study used placenta-derived MSCs (PD-MSCs, 10 patients) [27]. It is noteworthy that only 10 studies included a control group [15, 17, 20, 23, 25, 26, 28, 30, 35, 38]. Seven of them were divided into two groups on the basis of the patients’ willingness to serve as controls. The control group received the therapy with insulin intensification in addition to conventional therapy [17, 20, 23, 25, 28, 30, 35, 38]. In the other 3 studies, patients were randomly assigned to intervention where the patients received conventional therapy, and control arms where a sham procedure was applied to the patients [15, 26, 38].

### The outcome of stem cell therapy for T1DM

**Fig 2:**
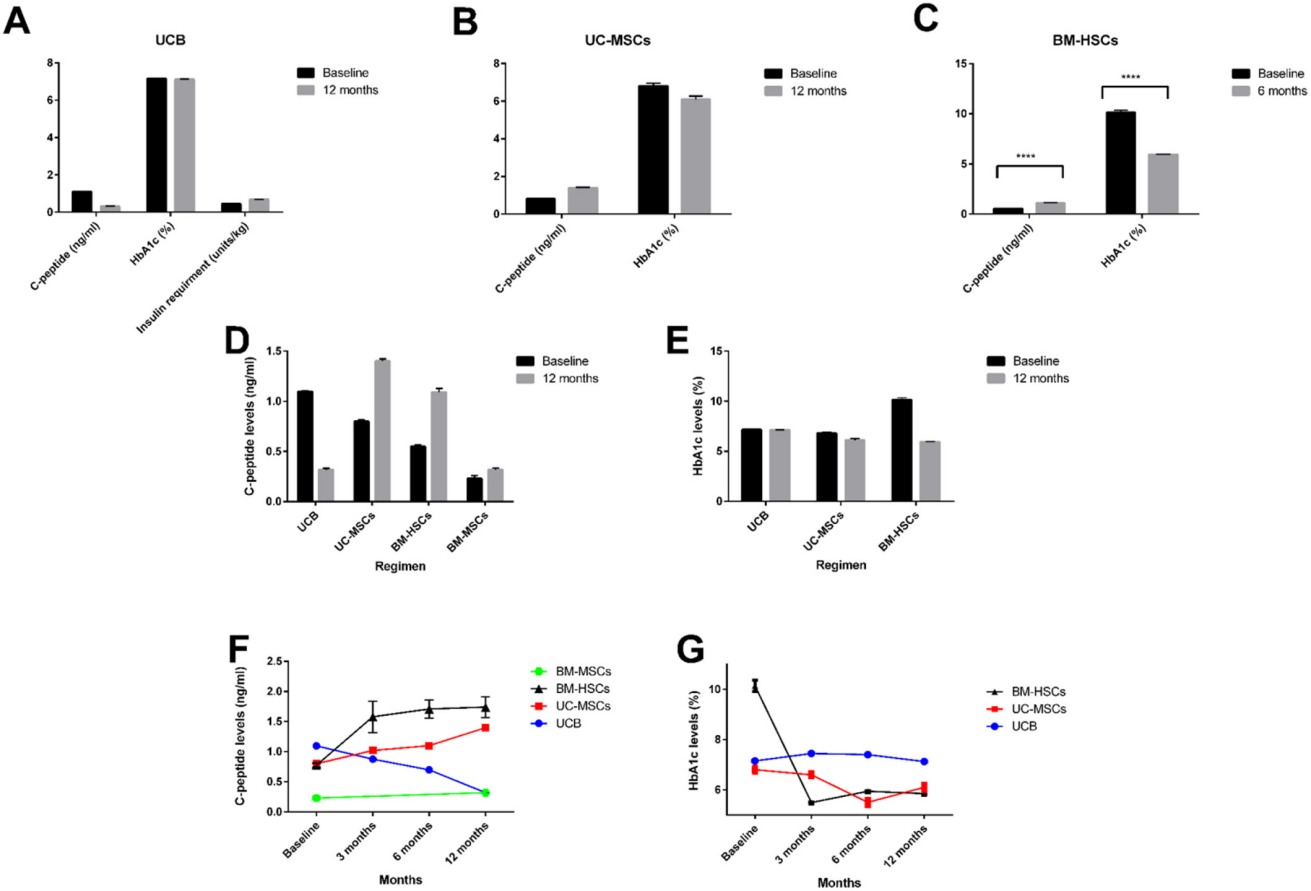
Stem cell therapy for type 1 DM. (A-C) Comparison of the outcome of different types of stem cells in T1DM by Intravenous administration of Umbilical Cord Blood (UCB, *n* = 61), Umbilical Cord-Mesenchymal Stem Cells (UC-MSCs, *n* = 15) and Bone Marrow Hematopoietic Stem Cells (BM-HSCs, *n* = 82) (D-E) Bar graphs depicting the change in C-peptide and HbA1c levels from baseline and 12 months after intravenous administration of different types of stem cells. (F-G) Line graphs showing changes in C-peptide and HbA1c levels over time at baseline, 3, 6 and 12 months after stem cell therapy in T1DM patients. All data are expressed as the mean ± SEM. **** P < 0.0001.

**Fig 3:**
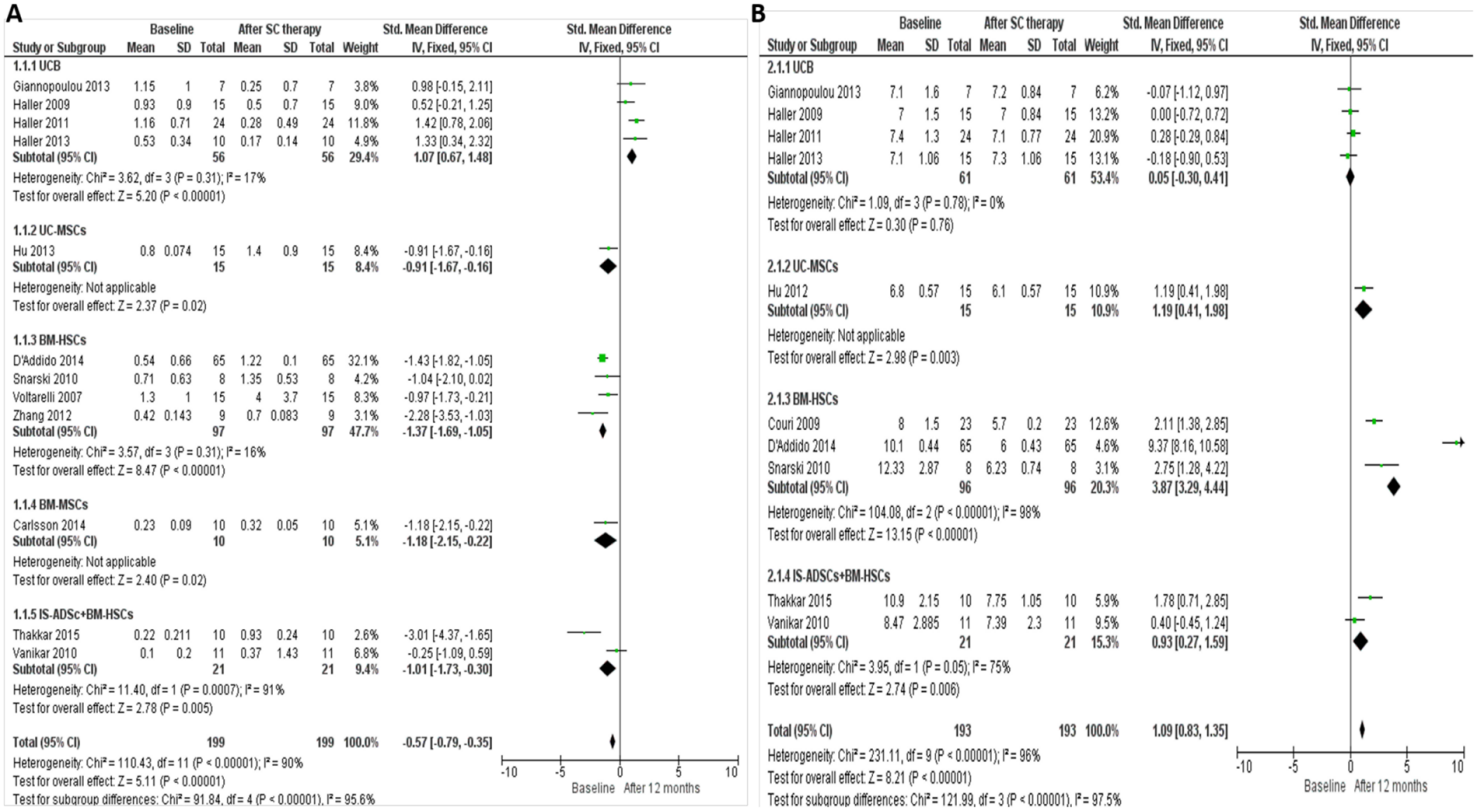
Forest plots showing the individual results of administrating different types of stem cells in T1DM. (A) Comparison of C-peptide levels in T1DM individuals from baseline and after 12 months of stem cells therapy (B) Comparison of HbA1c levels in T1DM individuals from baseline and after 12 months of stem cells therapy. The random-effects meta-analysis model (Mantel-Haenszel method) was used. The ends of the horizontal bars denote a 95% CI. The black diamond gives the overall mean difference for the combined results of all trials.

Stem cell therapy was carried out in 15 studies (300 patients, including 40 controls) with T1DM (Figs 2 and 3) as follows:

#### 1. Umbilical cord blood therapy

In 4 trials, autologous UCB cells with a mean dose of 1.49×10^7^ nucleated cells (mean number of CD34^+^ cells were 1.26×10^6^) were infused into 71 children (including 10 untreated controls who received insulin therapy only) with T1DM (the mean age was 5.28 years and mean history of diseases 6 months) [20, 22–24]. In these studies, the mean C-peptide peak levels at baseline in the treated group was 1.097±0.074 ng/ml compared to 0.32±0.09 ng/ml after 12 months of UCB infusion [95% CI 0.74614 to 0.80786, P<0.0001]. The mean HbA1c levels at baseline was 7.15±0.07% compared to 7.125±0.1% after 12 months of therapy [95% CI -0.00733 to 0.05733, P = 0.0866] (Fig 2). The mean daily insulin requirement was 0.447±0.07 units/kg at baseline compared to 0.68±0.014 units/kg after 12 months [95% CI -0.25190 to -0.21410, P<0.0001]. Although these results suggested that UCB infusion is without side effects and can be given safely to children with T1DM, the functional outcome of the treatment was uniformly negative as UCB infusion failed to improve C-peptide, HbA1c and insulin utilization levels at 12 months’ post-transplantation. The authors proposed that a possible explanation is that an insufficient number of cells carrying regenerative or immunoregulatory capacity were infused. To address this issue, efforts are underway to isolate and expand specific cell populations within UCB to augment their therapeutic potential (clinical trial reg. no. NCT01210664).

#### 2. Umbilical cord MSC therapy

A randomized controlled trial was carried out in 29 children with new onset T1DM (including 14 controls) with a mean age of 17.6 years to evaluate the long-term effects of UC-MSCs therapy [26]. The experimental group received a mean number of 2.6±1.2 × 10^7^ UC-MSCs, in addition to conventional insulin therapy. Control group received conventional insulin therapy alone. The mean C-peptide peak level at baseline was 0.8±0.074 ng/ml compared to 1.4±0.09 ng/ml after 12 months of therapy [95% CI -0.66162 to -0.53838, P<0.0001]. In the control group, the baseline levels were 0.89±0.39 ng/ml compared to 0.84±0.3 12 ng/ml at 12 months [95% CI -0.2102 to 0.3102, P = 0.69]. The mean HbA1c levels at baseline were 6.8±0.57% compared to 6.1±0.67% 12 months after treatment [95% CI 0.2348 to 1.1652, P = 0.7]. The baseline levels of the control group were 6.79±0.81% compared to 7.3±0.57% at 12 months [95% CI -1.0541 to 0.0341, P = 0.0650] (Fig 2). Out of the 15 treated patients, 4 became insulin independent, and the daily insulin requirements were reduced by more than 50% in another 7 for a period of 20–22 months. This suggested that UC-MSC therapy can provide sustained, long term functional improvement.

#### 3. Hematopoietic stem cell therapy

In a total of 6 studies involving 149 patients, granulocyte-colony stimulating factor (G-CSF)-mobilized CD34^+^ BM-HSCs retrieved from peripheral blood by leukapheresis were administered to patients with T1DM. The mean age of the patients was 18.8 ± 1.44 years, a mean history of disease of 7.14 months, and the mean follow up period was 21.48 ± 12.66 months. Autologous IV infusion of a mean dose of 6.99 ± 3.28 × 10^6^ cells/kg CD34^+^ BM-HSCs was carried out in 5 of the 6 studies involving 146 patients, and via liver puncture in the seventh [18, 19, 21, 28, 31, 36]. C-peptide levels were measured in 3 of the 6 studies (82 patients). The mean C-peptide peak levels significantly increased from 0.55±0.14 ng/ml at baseline to 1.09±0.34 ng/ml at 6 months after initiation of therapy [95% CI -0.6202 to -0.4598, P<0.0001]. HbA1c levels were determined in 3 of the 7 studies (96 patients). The mean level of HbA1c significantly decreased from 10.14±2.16% at baseline to 5.94±0.31% at 6 months’ post transplantation [95% CI 3.7607 to 4.6393, P<0.0001] (Fig 2).

Out of the 146 patients, 86 (58.9%) became insulin-free for a mean period of 16 months; and in 11 patients (7.53%), the insulin requirement was reduced by more than 50%. Out of the remaining 49 patients with poor prognosis, 39 (79.5%) had diabetic ketoacidosis (DKA). These data suggest that diabetic patients with DKA at diagnosis may not be good candidates for stem cell therapy. A possible explanation for this poor clinical response may be that DKA patients have very low β-cell reserve, as shown in several studies in which diabetic ketoacidosis at diagnosis was related to a decreased capacity for β cell recovery [39, 40].

Another important factor that influenced the treatment outcome was the time point of diagnosis of DM. Patients who received the therapy earlier after T1DM diagnosis (within 6 weeks) were twice as likely to achieve insulin independence over time than those with a later diagnosis (relative risk = 2.0, P = 0.0008), suggesting that early intervention with stem cell therapy achieves better outcome. 55 patients (34.1%) out of 161 reported mild to moderate side effects that shortly resolved, with the death of one patient as a result of *Pseudomonas aeruginosa sepsis*. With the exception of this infection related mortality, HSC therapy T1DM has shown no major side effects.

#### 4. Bone Marrow MSCs

A blind randomized trial was carried out to evaluate the effect of autologous BM-MSCs transplantation (the mean number of injected cells 2.75 × 10^6^/kg) in 20 T1DM patients (including 10 controls who received insulin therapy alone). The mean age of the patients was 24 ± 2 years and mean history of disease ≤ 3 months [17]. The baseline levels were similar amongst the two groups. The mean C-peptide peak level after 12 months in the control group was 0.29±0.04 ng/ml compared to 0.32±0.05 ng/ml in the MSC-treated group [95% CI -0.0725 to 0.0125, P = 0.1557]. The mean HbA1c levels at 12 months in the control group was 6.6±0.2% compared to 6.3±0.2% in the MSC- treated group [95% CI 0.112 to 0.488, P = 0.0035] (Fig 2).

Two additional studies reported transplantation of more than one type of stem cells into diabetic patients [32, 37]. In these studies, insulin-secreting adipose-derived MSCs (IS-AD-MSC) were co-transplanted along with BM-HSCs in 31 patients with T1DM. 10 patients received a mean dose of 2.65×10^4^/kg IS-AD-MSC co-transplanted with 2.14×10^4^/kg autologous HSCs and 21 patients received a mean dose of 2.07×10^4^/kg IS-AD-MSC co-transplanted with 6.6×10^3^/kg allogeneic HSCs via the portal system and thymic circulation. The group receiving the autologous transplantation showed better response to treatment in comparison to the group receiving allogeneic treatment (Fig 3).

### The outcome for stem cell therapy for T2DM

**Fig 4:**
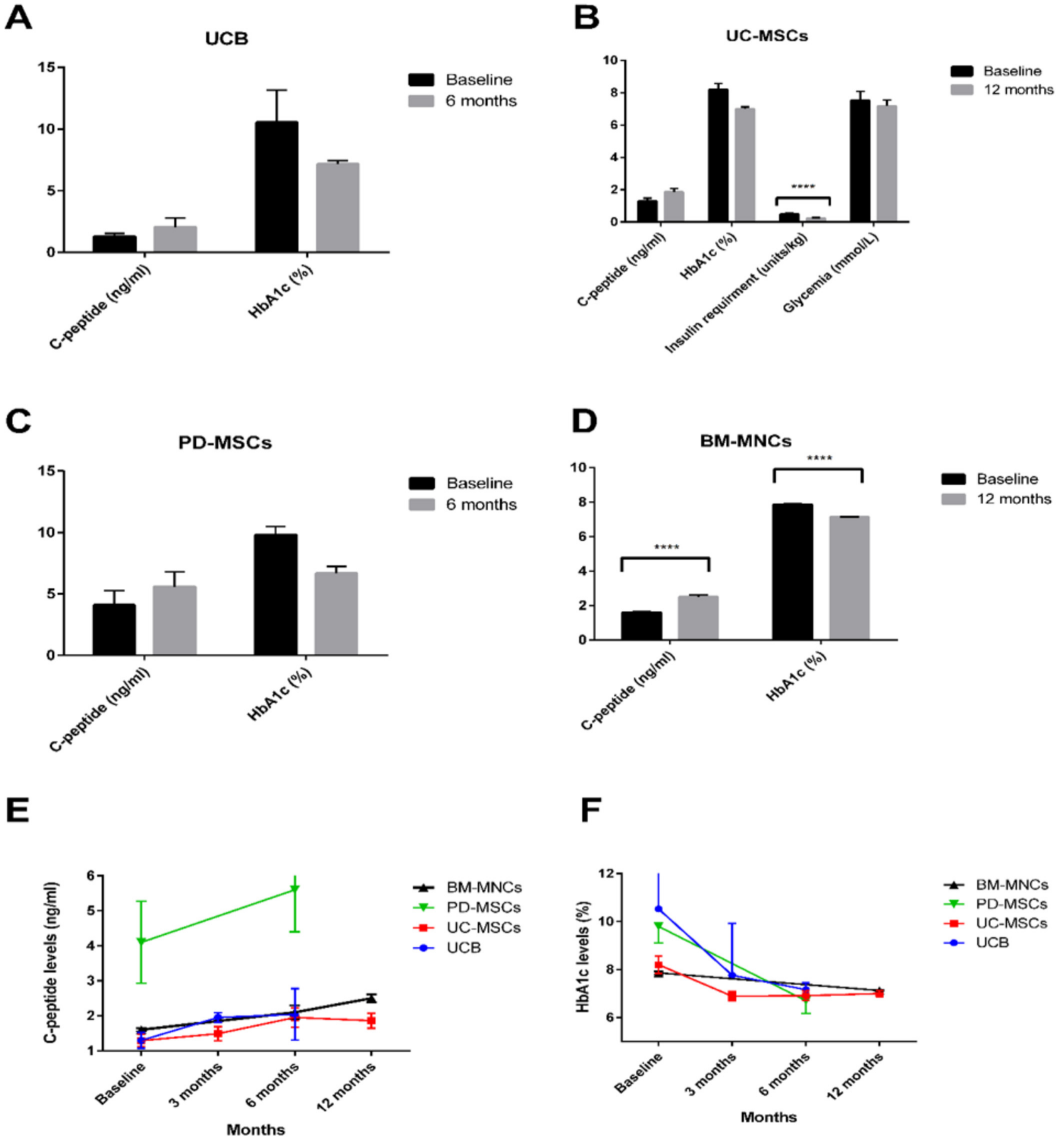
Stem cell therapy for type 2 DM. (A-D) Bar graphs depicting the change in C-peptide and HbA1c levels from baseline and 12 months after administration of different types of stem cells. UCB and BM-MNCs were injected intra-pancreatically *(n* = 3 and *n* = 107, respectively), while UC-MSCs and PD-MSCs were injected intravenously *(n* = 22 and *n* = 10, respectively). (E-F) Line graphs showing changes in C-peptide and HbA1c levels over time at baseline, 3, 6 and 12 months after stem cell therapy in T2DM patients. All data are expressed as the mean ± SEM. **** P < 0.0001.

**Fig 5:**
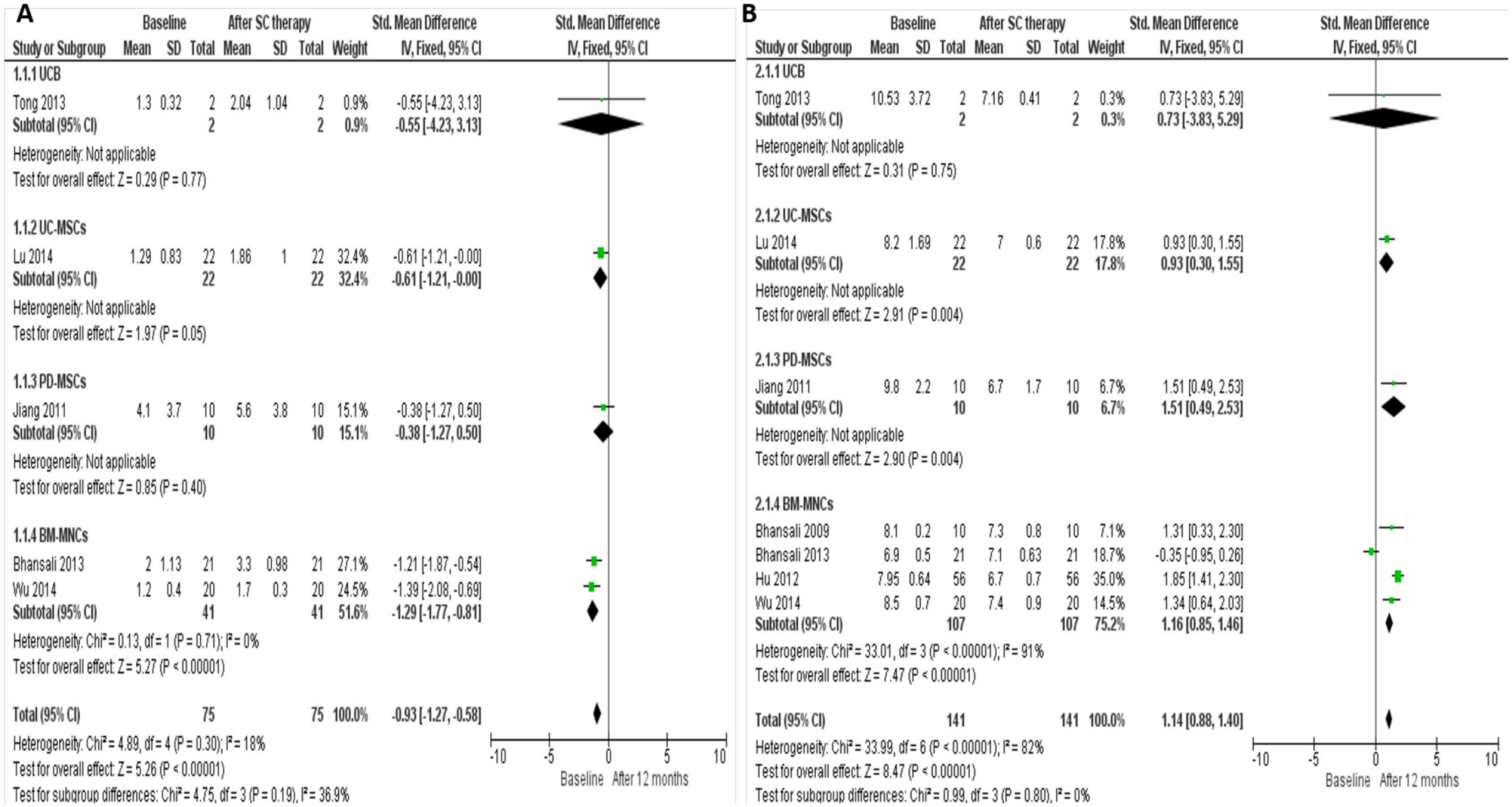
Forest plots showing the individual results of administrating different types of stem cells in T2DM. (A) Comparison of C-peptide levels in T2DM individuals from baseline and after 12 months of stem cells therapy (B) Comparison of HbA1c levels in T2DM individuals from baseline and after 12 months of stem cells therapy. The random-effects meta-analysis model (Mantel-Haenszel method) was used. The ends of the horizontal bars denote a 95% CI. The black diamond gives the overall mean difference for the combined results of all trials.

Stem cell therapy for T2DM was carried out in 7 studies involving 224 patients (including 92 controls) (Figs 4 and 5) as follows:

#### 1. Umbilical cord blood therapy

Tong et al. [33] utilized autologous UCB therapy (the mean dose of 5.29×10^9^ nucleated cells; and the mean number of CD34^+^ cells was 2.88×10^6^) via intra-pancreatic infusion. The mean C-peptide peak level before therapy was 1.30±0.32 ng/ml; and was elevated to 2.04±1.04 ng/ml at six months after therapy [95% CI -2.4842 to 1.0042, P = 0.3041]. The mean HbA1c level was 10.53±3.72% at baseline, and was reduced to 7.16±0.41% after 6 months of infusion [95% CI -2.6292 to 9.3692, P = 0.1939] (Fig 4).

#### 2. Umbilical cord MSC therapy

A mean dose of 1×10^6^/kg UC-MSCs was infused intravenously followed by an intrapancreatic infusion in 22 patients. The mean age of the patients was 52.9 ± 10.5 years, and the mean history of T2DM was for 8.7 ± 4.3 years [29]. The mean C-peptide peak level at baseline was 1.29±0.83 ng/ml. It reached its peak at 6 months to become 1.95±1.3 ng/ml, and at 12 months it was slightly reduced to 1.86±1.0 ng/ml [95% CI -1.3236 to 0.0036, P = 0.0512]. The mean HbA1c levels at baseline was 8.20±1.69%. It reached its maximum decline at 6 months to become 6.91±0.96%, after which it was slightly elevated to 7.0±0.6% at 12 months [95% CI 0.4284 to 1.9716, P = 0.0031] (Fig 4). The mean daily insulin requirement declined from 0.49±0.22 units/kg at baseline to 0.23±0.19 units/kg 12 months after therapy [95% CI 0.1349 to 0.3851, P<0.0001]. These data suggested that UC-MSC treatment provided a long lasting therapeutic effect. In this study, self-resolved mild to moderate side effects were reported in only 3 patients.

In one trial, a mean number of 1.35×10^6^/kg PD-MSCs were transplanted into 10 patients [27]. The mean C-peptide peak level was 4.1±3.7 ng/ml at baseline, and increased to 5.6±3.8 ng/ml after 6 months of therapy [95% CI -5.024 to 2.024, P = 0.3829]. The mean HbA1c level was 9.8±2.2% at baseline compared to 6.7±1.7% after 6 months [95% CI 1.253 to 4.947, P = 0.0024]. The mean daily insulin requirement levels decreased from 63.7±18.7 IU at baseline to 34.7±13.14 IU after 6 months [95% CI 13.8158 to 44.1842, P = 0.0008].

#### 3. Bone marrow mononuclear cell therapy

In 4 studies, Intra-pancreatic Infusion of autologous BM-MNCs retrieved by aspiration from posterior iliac crest was carried out in 189 T2DM patients (including 82 controls who received insulin therapy and a placebo therapy). The mean dose of nucleated cells of 17.29 × 10^8^ cells/kg (mean number of CD34^+^ cells was 3.15×10^6^) [15, 16, 25, 35]. The mean age of patients was 53.7±3.5 years. C-peptide levels were described in 2 of the 4 studies (41 patients). The mean C-peptide peak levels significantly increased from baseline level of 1.6±0.5 ng/ml to 2.5±1.13 ng/ml 12 months’ post transplantation [95% CI -1.2840 to -0.5160, P<0.0001]. The levels of HbA1c were determined in all of the 4 studies involving 107 patients. The mean HbA1c peak levels significantly decreased from a baseline level of 7.86±0.68% to 7.125±0.30% 12 months after therapy [95% CI 0.59337 to 0.87663, P<0.0001] (Fig 4). Of the 107 patients who received BM-MNC transplantation, 18 (16.8%) became insulin independent, and 47 (43.92%) showed more than 50% reduction of insulin requirement. Eight patients only (7.4%) reported mild adverse effects of abdominal pain and nausea, indicating the relative safety of this form of therapy.

## Discussion

Along with the obesity epidemic, the incidence of DM is rapidly increasing worldwide. Considering that insulin administration only postpones complications, new approaches to cure diabetes or provide sustained therapeutic outcome are needed. To date, approaches that include islet cell transplantation, pancreas transplantation, and administration of anti-CD3 mAb have been approved for clinical use [41–43]. According to the United Network for Organ Sharing (UNOS) Data Registry Analysis, pancreas transplantation accomplished insulin independence in 60% of subjects at 4 years after the transplant. However, this surgical approach still suffers significant mortality (78% survival at 1 year) [44]. Results from the Collaborative Islet Transplant Registry (CITR) indicate that 44% of recipients were insulin independent at 3 years post-transplant, from 2007–2010, as compared to 27% of clinical islet transplant recipients in 1999–2002 [45]. This increase in success rates, can be attributed to improvements in immunosuppression strategies. On the other hand, anti-CD3 mAbs therapy, although considered moderately safe, accomplished insulin independence in only 5% of subjects at 2 years’ follow-up [43]. Despite this modest outcome, islet cell transplantation has been successful in halting the progression of both short-term and long-term complications of DM [46, 47]. Islet transplantation however suffers many challenges, especially the limited supplies of donors and their high variability [3, 48].

The present meta-analysis is, to our knowledge, the first attempt to systematically collect all available evidence and critically assess and quantify the safety and efficacy of stem cell therapy for DM. We include all types of stem cell therapies applied in both T1DM and T2DM patients.

Our analysis indicates that the type of injected cells is of prime importance in the outcome of therapy. Intravenous administration of CD34^+^ BM-HSC, collected by leukapheresis of peripheral blood after G-CSF mobilization, showed the best outcome. As 58.9% of treated T1DM patients became insulin independent for a mean period of 16 months. An additional 7.53% showed more than 50% reduction of insulin requirement, suggesting the efficacy of CD34^+^ BM-HSCs in treating DM. Intravenous infusion of UCB on the other hand, although containing similar numbers of CD34^+^ cells shown in BM-HSC transplantation, failed to improve C-peptide levels, HbA1c levels, and insulin utilization levels in T1DM patients. However, this data is based on small number of trials, and need confirmation in larger randomized trials.

The discrepancy between the promising therapeutic outcomes using marrow CD34^+^ HSCs compared to the poor outcome of cord blood cells is puzzling, considering that both transplants include hematopoietic progenitors. In the mobilized bone marrow stem cells, G-CSF was used to stimulate endogenous stem cells. Stem cells were then freshly isolated, and re-infused into the same patient. Cord blood cells on the other hand were not similarly purified CD34^+^ population, but included a mixture of cells at different stages of differentiation, and although they were also autologous, they were frozen-thawed. These data may indicate that the number, source, and freshness of CD34^+^ stem cell preparation all contribute to the therapeutic effectiveness of the cell therapy. Evidently, differences between marrow cells and cord blood cells in homing, engraftment and differentiation potential may have contributed to this disparate outcome, and as mentioned, larger numbers of randomized trials are necessary for accurate comparison.

Although the majority of experimental research focus on the use of MSCs in DM [49, 50]. Mobilized CD34^+^ BM-HSCs showed better outcomes than MSCs from both marrow and umbilical cord in improving C-peptide and HbA1c levels, although the latter also showed effective therapeutic outcome. These data confirm that the effectiveness of stem cell therapy in DM multifactorial, and is probably also dependent on the previously described immunoregulatory properties of CD34^+^ cells, regeneration of a naive immune system from autologous HSCs, and the possibility of regeneration of β-cells from autologous marrow stem cell. Since most experimental research focus on differentiation of stem cells into insulin-producing cells as the goal of diabetes therapy, the present study suggests a different paradigm and proposes that studies on therapeutic effects of stem cells in DM should consider remodeling of the diabetic microenvironment, in addition to islet cell replacement as an effective approach to treatment.

Infusion of UC-MSCs achieved better outcome than BM-MSCs in improving C-peptide levels in T1DM patients (P<0.0001 and P = 0.1557, respectively). While Infusion of BM-MNCs provided better outcome compared to UC-MSCs in improving C-peptide levels in T2DM patients (P<0.0001 and P = 0.0512, respectively). No conclusive recommendation could be deduced from these data as they were based on small number of trials, and need confirmation in larger randomized trials.

Another factor that contributed to the better outcome of stem cell therapy and improved glycemic control was the number of the injected CD34^+^ cells. This number was positively correlated with levels of C-peptide and HbA1c and with reduced doses of insulin requirement [28]. Furthermore, the numbers of infused CD34^+^ cells were positively correlated with the levels of anti-inflammatory cytokines such as IL-4, IL-6 and TGF-β but negatively correlated with pro-inflammatory cytokines such as TNF-α [28].

Our data show that the efficacy and functionality of the injected stem cells were also dependent on the route of administration. The intra-pancreatic infusion of UCB through the dorsal pancreatic artery showed better results in improving the levels of C-peptide and HbA1c, than IV perfusion. Interestingly, supportive animal data showed that after systemic IV perfusion, high concentrations of cells could be observed in the lung and liver, but not in the pancreas [51]. In addition, intra-pancreatic delivery of human UCB in mice resulted in the engraftment of a higher number of infused cells, leading to islet regeneration and increased insulin release [52]. This approach however can be challenging due to the variability in the anatomical position of the dorsal pancreatic artery [53, 54].

It is noteworthy that only 21.72% of patients receiving stem cell transplantation for either T1DM or T2DM showed adverse effects. Stem cells thus seem to be a safer form of transplantation therapy for the treatment of DM, compared to whole organ and islet transplantation. However, stem cell therapy still caused some severe adverse effects, mostly as a result of the administration of a high-dose of the immunosuppressive regimen. Although these side effects were resolved shortly after stem cell therapy and subsequent immune reconstitution, modification in the therapy needs to be considered. Better-tolerated, lower doses of immunosuppressive medications along with stronger prophylaxis against infection seem to be critical for a better therapeutic outcome.

It is noteworthy that all trials using mobilized HSCs were in T1DM patients, and all T2DM patients who received BM-MNCs were transplanted via the intrapancreatic route. This invasive method of therapy was required in T2DM because of the chronic nature of the disease and their poor response to G-CSF mobilization [55]. Although it showed the best therapeutic outcome, CD34^+^ cells mobilization in diabetic patients needs to be considered cautiously, as mobilization of both stem cells and proangiogenic cells seems to be impaired in diabetics [55]. Alternative approaches for stem cell mobilization such as the use of the C-X-C chemokine receptor type 4 inhibitor (Plerixafor), which is now applied in several hematopoietic disorders [56, 57], may be worth consideration in diabetic patients as well, especially the chronic T2DM [58]. It is also noteworthy that the insulin-independence by transplantation of mobilized CD34^+^ BM-HSCs was achieved in recently diagnosed, early stage DM patients, while islet transplantations in the CITR trials achieved this dependency in advanced patients. The success of islet transplantation, in part, can be attributed to shifts in immunosuppression strategies to a better combination that considers the immune status and fragility of the patients [59]. However, in the CD34^+^ BM-HSCs transplantation, immunosuppression was achieved with administration of high dose cyclophosphamide and antithymocyte globulin, which are potentially toxic immunosuppressive agents that cause long term complications, and lack the selective immune suppression, and fewer complications were achieved with the multifactorial regimens followed prior to islet transplantation [60]. In addition to immune therapy, the role of stem cell therapies in DM to establish long term insulin-independency could be more attainable with the use of better, safer, and more effective immunosuppressive regimen, and anti-inflammatory agents, paired with autologous stem cell transplantation.

Although this meta-analysis demonstrated that autologous stem cell transplantation can be considered a safe and effective approach for treatment of many DM patients, it also has notable limitations. One of the most important limitations is that some of the cases covered in this meta-analysis have been evaluated in a single study with low statistical power. In some studies, low number of patients enrolled, not sufficient for significant interpretation. The total sample size was not large and the follow-up time was not adequately long. Moreover, the number of trials was relatively small, and more accurate recommendations could be made when analyzing a larger cohort. Altogether, these shortcomings reflect the paucity of the available published data on this very important and much anticipated form of cell therapy and indicates the large gap between the preclinical and clinical studies. Moreover, to determine the proper type of stem cells for therapy, route of injection, etc., experimental studies that address the interactions between stem cells and islet cells, and the exact molecules and pathways involved in their therapeutic effect are still needed.

On the basis of our study, we conclude the following: (1) remission of DM is possible following stem cell therapy; (2) stem cell transplantation can be a safe and effective approach for therapy of DM; (3) available data from these clinical trials indicate that the most promising therapeutic outcome was shown in mobilized marrow CD34^+^ HSCs; (4) patients with previously diagnosed diabetic ketoacidosis are not good candidates for the applied approaches stem cell therapy; (5) stem cell therapy at early stages after DM diagnosis is more effective than intervention at later stages; and (6) well-designed large scale randomized studies considering the stem cell type, cell number, and infusion method in DM patients are urgently needed.

## Supporting Information

S1 PRISMA Checklist PRISMA Checklist. (DOC)
